# Applying Educational Data Mining to Explore Viewing Behaviors and Performance With Flipped Classrooms on the Social Media Platform Facebook

**DOI:** 10.3389/fpsyg.2021.653018

**Published:** 2021-04-29

**Authors:** Yu-Sheng Su, Chin-Feng Lai

**Affiliations:** ^1^Department of Computer Science and Engineering, National Taiwan Ocean University, Keelung City, Taiwan; ^2^Department of Engineering Science, National Cheng Kung University, Tainan, Taiwan

**Keywords:** educational data mining, social media platform, facebook, viewing behaviors and performance, learning performance

## Abstract

In recent years, learning materials have gradually been applied to flipped classrooms. Teachers share learning materials, and students can preview the learning materials before class. During class, the teacher can discuss students' questions from their notes from previewing the learning materials. The social media platform Facebook provides access to learning materials and diversified interactions, such as sharing knowledge, annotating learning materials, and establishing common objectives. Previous studies have explored the effect of flipped classrooms on students' learning engagement, attitudes, and performance. In this paper, we apply educational data mining to explore the relationship between students' viewing behaviors in accessing learning materials and their performance in flipped classrooms. The participants are classified into an experimental group and a control group to engage in flipped classroom activities. The experimental group uses the social media platform Facebook for flipped learning, and the control group uses a learning management system for flipped learning. The results show that there is a significant difference in the learning performance between the two groups, with the average score of the experimental group being higher than that of the control group. Furthermore, we find that the viewing behaviors and performance of the students within the experimental group differ significantly.

## Introduction

With the popularity of flipped classrooms, most teachers have begun to implement the flipped learning approach, which allows students to preview learning materials before class. Flipped classrooms are a student-centered method; that is, students spend more time on and assume more responsibilities for preclass learning. Flipped classrooms are mainly divided into two modes: the preclass learning mode and the in-class learning mode (Kim et al., 2014; Su et al., 2014; Brinton et al., 2015; de Barba et al., 2016; Lai and Hwang, 2016; Li and Tsai, 2017; Long et al., 2017; Sun et al., 2017; Lee, 2018). Before class, the teacher applies the preclass learning mode, and students preview learning materials and complete quizzes to enhance their prior knowledge. In class, based on students' prior knowledge, the teacher adopts the in-class learning mode to develop students' new knowledge about the new learning materials. Although learning materials are the most commonly used materials in flipped learning, they have two disadvantages. First, not all students want to watch learning materials before class. Kim et al. (2014) observed that 25% of students did not preview learning materials. Second, learning materials are presented with multimedia information. Students preview learning materials and processes and digest the content of the learning materials through the working memory in their brains, which could lead to excessive learning and cognitive load (Mayer, 2002; Manley and Urness, 2014; Lin et al., 2015).

To address the above problems, most teachers have students ask questions and participate in discussions in learning management systems to understand the flipped learning situation of the students (Henrie et al., 2015; O'Flaherty and Phillips, 2015). O'Flaherty and Phillips (2015) indicated that to build students' prior knowledge, they need to complete the previewing learning tasks, and the task content is the learning materials made by teachers such as textbooks and videos. However, the teacher does not know whether students take the learning tasks seriously when they complete them. Previous researchers (McFadden et al., 2014; Cummins et al., 2015; Chiu et al., 2016) have developed extensive annotation systems. Students can use such annotation systems to help teachers understand the problems that they encounter; for example, students can annotate learning materials to reflect on their learning difficulties. In addition to the annotation function, Cummins et al. (2015) developed an interactive teaching system that allows students to add questions as part of their annotation. Chiu et al. (2016) also developed an interactive teaching system in which students can use arrows, rectangles, and circles to mark the learning material content at any point during the video. The results show that these systems can help students have a strong memory of the marked parts of the learning material and improve their learning performance.

Traditional learning management systems have some limitations. For example, Wopereis et al. (2008) observed that students only logged into the learning management system to preview learning materials at certain moments and that learning materials were available for only one semester on the learning management system. In recent years, social media systems have gradually been integrated into e-learning systems. Kim et al. (2014) applied flipped classrooms in engineering courses. Students were required to read the learning materials on the social media platform Facebook before class and could discuss the learning materials on the Facebook discussion board. Social media platforms provide access to learning materials and offer diversified multimedia interactions, for example, sharing knowledge, annotating multimedia, and establishing common learning objectives (Sherry et al., 2011; Hou et al., 2013; Kim et al., 2014; Lin et al., 2014; Yilmaz and Baydas, 2017; Su et al., 2019; Su and Chen, 2020). Yu et al. (2010) found that the appropriate usage of the social media platform Facebook to preview learning materials helped enhance students' preclass learning motivations. Su and Chen (2020) asserted that Facebook has intensive social interactions, including exchanges of different knowledge, backgrounds and perspectives, and a relaxing atmosphere for social interactions. Therefore, the social media platform Facebook supports e-learning systems to help students maintain preclass learning practices. Furthermore, students do not have to create a new account for each course; thus, the contact and discussion among students do not end when the class ends (Hou et al., 2014; Mendoza et al., 2015).

Educational data mining includes statistics, visualization, classification, clustering, associative analysis, anomaly detection, and text mining (Bakeman et al., 2009; Baker and Yacef, 2009; Baker, 2010; Lu et al., 2018; Su et al., 2019). Data mining is used to analyze the digital content player system logs that are generated during flipped learning activities, such as student equipment usage and conversation records, to obtain useful information and share information with teachers and creators to improve future e-learning platforms. Previous studies (Jeong, 2003; Brooks et al., 2014; Giannakos et al., 2015a,b; Kuo et al., 2015; Park et al., 2017; Su and Wu, 2020; Su et al., 2020) have applied educational data mining to analyze the time that students spend viewing learning materials to determine viewing behaviors. Park et al. (2017) used clickstream data to detect mouse changes in students' viewing behaviors. They applied educational data mining to identify students' mouse changing patterns and explained how these changes are related to student viewing behaviors.

To explore the behavioral patterns of viewing learning materials, previous researchers (Bouchet et al., 2012; Peckham and McCalla, 2012; Sinha et al., 2014; Brinton et al., 2015; Liu and Xiu, 2017) have explored the system logs that students use to read learning materials; continuous viewing behaviors indicate that students are performing certain learning activities. Moreover, the system logs allow the identification of the meaningful viewing of behavioral events, for example, repetition, skipping, rewinding, and slow watching. The findings indicated that meaningful viewing behaviors have a strong connection with students' learning performance and can be applied to understand students' learning performance. Bouchet et al. (2012) analyzed clicking data from multimedia players to mine better viewing behavioral models to predict students' future viewing behaviors and performance. Peckham and McCalla (2012) used educational data mining to mine student behavior patterns in viewing learning materials, and they showed how these viewing patterns are related to viewing performance.

In this paper, this study applied educational data mining to explore the relationship between students' viewing behaviors in accessing learning materials and their performance in flipped classrooms. The experimental subjects were 56 students, who were divided into an experimental group and a control group to engage in flipped learning activities. The experimental group used the social media platform Facebook for flipped learning, and the control group used a learning management system for flipped learning. By analyzing the questionnaire data, learning performance, and system logs, we explored the difference in the viewing behaviors between the two groups. In terms of viewing behaviors, we analyzed the system logs of the two groups to determine the differences in the viewing behaviors and performance between the two groups. Therefore, this study proposes the following three research questions.

1: What are the differences in learning achievements between the two groups?
2: What are the differences in the system user acceptance between the two groups?
3: What are the differences in viewing behaviors, as an indicator of students' performance, between the two groups?

## Methodology

### Participants

The participants comprised a total of 56 freshmen with an average age of 19 years from a college in northern Taiwan. Two classes were assigned as the experimental group, while the other class was the control group, with student numbers of 27 and 29, respectively. The two groups were taught by different teachers at different times. All students were equipped with basic computer skills. Students in the experimental group used the social media platform Facebook combined with flipped learning. Students in the control group used a learning management system combined with flipped learning.

### Learning Materials

The learning materials were selected from “*Cloud Technology and Internet Services*” (Computer Skills Foundation, 2013). The learning goal of this course was to develop the concepts of related technologies. To facilitate students' understanding of the course subject, the teacher designed three instructional units, including the concept of virtualization technology, the basic operation of Docker virtualization tools, and skills with a demonstration of practical development.

### Procedures

The experimental activities lasted 5 weeks and were conducted for 50 min each. Before the experimental activities, the teacher presented the operating steps to use the social media platform Facebook or the learning management system in flipped learning. Next, the teacher confirmed whether each student could use these systems to preview the learning materials. The students practiced how to use the systems to preview learning materials. Finally, the teacher announced that flipped learning assignments needed to be completed each week and explained the grading standards and requirements for the preview assignments.

During week 3 of the flipped classroom activities, the teacher covered course topics such as the concept of cloud technologies, basic operations of Docker, and skills of practical development. The students used the systems to complete flipped learning assignments before class. The students answered the questions from the teacher by repeatedly watching the learning materials on the social media platform Facebook or the learning management system. The students sought assistance from the teacher through these systems and learned by constantly asking questions related to the new learning material and cultivating their critical thinking ability. In class, the teacher discussed the students' questions and addressed the problems that they encountered during flipped learning. The teacher gave tips in advance to the students who had encountered problems to reduce learning frustration.

After the experimental activities were completed, the students in the experimental and control groups finished the posttest and a system user acceptance questionnaire. The posttest was conducted to measure the students' learning achievements. This questionnaire surveyed the students' acceptance of using the systems. Finally, summarizing the system logs helped us further explore how the viewing behaviors of the two groups affected their learning performance.

### Instruments

The posttest was designed by two senior teachers with more than 3 years of teaching experience in “Cloud Technology and Internet Services” based on the TQC Cloud Technology and Network Services Assessment (Computer Skills Foundation, 2013). We used the posttest to evaluate the learning achievements of the students. The test included 20 multiple-choice questions. Each question had four response options, and the total number of points was 100. The discrimination and difficulty of the posttest questions were 42 and 52%, respectively.

Based on Lund's questionnaire (Lund, 2001), we developed a system user acceptance questionnaire. This measure of usability has been shown to have good reliability and good validity and assessed whether users think a system is useful and easy to use and whether they want to use it in the future. Based on Lund's questionnaire, we designed a system user acceptance with a total of six items. Each item of this questionnaire was designed on a five-point Likert scale. The Cronbach α of the questionnaire was 0.83, and the Cronbach α of the two dimensions, i.e., usefulness and ease of use, were 0.86 and 0.80, respectively, which demonstrates good internal consistency and reliability.

### Data Collection and Analysis

All students in both groups were evaluated with the system user acceptance questionnaire and the posttest. Moreover, we collected data on the learning material viewing behaviors of both groups of students who used the different systems. To analyze the learning material viewing behaviors, we defined four indicators to represent students' viewing behaviors.

The definition of the viewing time indicator is the “total time that the multimedia material was viewed.” The definition of the active viewing time indicator is the “total time that the mouse pointers of the learners were concentrated on the learning material.” The definition of the viewed amount indicator is the “total amount of multimedia material viewed by the student.” The definition of the actively viewed amount indicator is the “total amount of multimedia material viewed while the mouse points of the learners were concentrated on the material.”

The values of the four indicators were calculated based on the students' learning material viewing behaviors. The operational definitions of the four indicators, especially regarding the students' mouse cursor activity, focused on the learning materials. Next, the k-means clustering method was applied to these indicators, which generated three clusters. Cluster 1 comprised students with the minimum time spent viewing the learning material, cluster 2 comprised students with an intermediate time spent viewing the learning material, and cluster 3 comprised students with the longest time spent viewing the learning material. Finally, we compared the three clusters regarding the students' behaviors and learning performance. We applied the Kruskal–Wallis test to analyze the statistical significance of the differences among the three clusters in the dependent variable. The pairwise Mann–Whitney U test was then used for the *post-hoc* test.

## Results

### Analysis of Learning Achievements

The posttest was applied to assess whether there were significant differences in learning achievements between the experimental group and the control group. The results of the independent-samples *t*-test of learning performance of the two groups are shown in Table 1; the *t* value is 1.921, the Cohen d value is 0.755, the effect size value is 0.386, and the *p*-value of the two-tailed test is 0.032 (*p* < 0.05). We can see that the result consists of small effects, which indicates that there was a significant difference in the learning achievements between the two groups, with the average score of the experimental group [mean = 85.132, standard deviation (SD) = 7.191] being higher than the average score of the control group (mean = 78.312, SD = 10.243).

**Table 1:** Independent-samples *t*-test of the learning achievements of the two groups.

**Group**	**N**	**Mean**	**SD**	***t***	***p***
Experimental group	27	85.132	7.191	1.921	0.032^*^
Control group	29	78.312	10.243		

* *p < 0.05*.

### Analysis of System User Acceptance

In Table 2, the analytical results of the questionnaire show no significant differences in the system user acceptance between the two groups; the *t* value is −0.536, and the *p*-value of the two-tailed test is 0.362. The mean of the experimental group was higher than 4.0, which indicates that the experimental group students had a high acceptance of the use of the social media platform Facebook for flipped learning. However, the mean of the control group was 3.7, which indicates that the students had a low acceptance of the use of the learning management system for flipped learning.

**Table 2:** Independent-samples *t*-test of the system user acceptance of the two groups.

**Group**	**N**	**Mean**	**SD**	***t***	***p***
Experimental group	27	4.028	0.5131	−0.536	0.362
Control group	29	3.732	0.6231		

* *p < 0.05*.

### Analysis of Viewing Behaviors and Performance

#### Descriptive Statistics

The total length of time for viewing the learning materials was 15 min 43 s. Table 3 illustrates the means and SDs for the viewing behaviors of the two groups, including the time spent viewing the learning materials. The students' learning material viewing behaviors were assessed according to the following four indicators: the viewing time, active viewing time, viewed amount, and actively viewed amount. Although the SDs of the viewing time and the active viewing time of the two groups differed greatly, the SDs of the viewed amount and actively viewed amount differed less. The shortest viewing time of the students in the two groups was 782 s, and the completion rate of all students was higher than 83%, which indicates that the students in the two groups almost completed all of the flipped learning tasks. However, the viewing behaviors of the students in the two groups were very different.

**Table 3:** Descriptive statistics for the viewing behaviors of the two groups.

**Groups**	**Indicators**	**Min**	**Max**	**Mean**	**SD**
Experimental group	Viewing time (s)	936	3,832	1,632.38	732.35
Control group		922	3,928	1,537.17	823.38
Experimental group	Active viewing time (s)	326	2,832	1,213.83	536.23
Control group		254	2,968	1,173.78	693.56
Experimental group	Viewed amount (s)	873	943	903.32	53.37
Control group		782	943	926.06	44.86
Experimental group	Actively viewed amount (s)	319	943	836.38	182.73
Control group		209	943	785.00	232.87

According to the statistics, the number of times that the students spent viewing learning materials (viewing time) was 4.27 for the experimental group and 3.68 for the control group. The number of times that the students actively viewed the learning materials (active viewing time) was 1.363 for the experimental group and 1.124 for the control group. The minimum length of the viewed amount was 873 s for the experimental group and 782 s for the control group. The minimum length of the actively viewed amount was 319 s for the experimental group and 209 s for the control group. The students in the two groups focused on viewing the learning materials; on average, they concentratedly watched 87% of the learning materials.

#### Analysis of Viewing Behaviors

Based on the four indicators, we applied educational data mining to divide the students with similar watching behaviors into the same category. Sinha et al. (2014) suggested that the four features could be transformed to minimize the clustering bias before conducting the k-means clustering method. First, the non-normalized data of the viewing behavior patterns were converted into normalized data to reduce the clustering bias. Second, each iteration was refined for the appropriate solutions by measuring the distance between the centroid points and the related data points. Finally, we designated the lowest, intermediate, and highest time (33.33%) durations for cluster 1 with the experimental group students (*N* = 9, viewing time = 2.00, active viewing time = 1.20, viewed amount = 1.80, actively viewed amount = 1.20) and the control group students (*N* = 9, viewing time = 2.10, active viewing time = 1.40, viewed amount = 1.80, actively viewed amount = 1.40), cluster 2 with the experimental group students (*N* = 9, viewing time = 1.00, active viewing time = 1.60, viewed amount = 1.60, actively viewed amount = 2.20) and the control group students (*N* = 9, viewing time = 1.46, active viewing time = 1.83, viewed amount = 1.83, actively viewed amount = 2.60), and cluster 3 with the experimental group students (*N* = 9, viewing time = 2.63, active viewing time = 2.75, viewed amount = 2.00, actively viewed amount = 2.38) and the control group students (*N* = 11, viewing time = 3.23, active viewing time = 2.955, viewed amount = 2.20, actively viewed amount = 2.57).

To clearly understand the learning material watching behaviors of the students in the two groups, we compared the three clusters to analyze with Kruskal–Wallis tests the amount of time that they spent viewing multimedia material and the number of times that they viewed the learning materials. To perform cross-comparisons of the pairs of clusters, we used pairwise Mann–Whitney U tests to conduct a *post-hoc* analysis. The experimental group students in the three categories exhibited significantly different behavior patterns when viewing the learning materials [x^2^ (2, *N* = 27) = 5.312, *p* = 0.042].

#### Analysis of Learning Performance Among Clusters

We applied the Kruskal–Wallis test to examine whether the three clusters differed in their learning achievements in the experimental and control groups. As shown in Table 4, the results for the experimental group revealed that there was a significant difference between clusters 1 and 2 (*U* = 12.400 *z* = 1.21, *p* = 0.021, *r* = 0.43) and between clusters 2 and 3 (*U* = 24.300, *z* = 1.102, *p* = 0.012, *r* =0.46). This finding shows that the learning performance in the experimental group was significantly higher for clusters 1 and 3 than for cluster 2. However, the results of the control group demonstrate that there was no significant difference among the three clusters.

**Table 4:** Learning achievements among the three clusters.

**Group**	**Cluster 1 (C1)**		**Cluster 2 (C2)**		**Cluster 3 (C3)**		**Kruskal–Wallis test**	***Post-hoc* test**
	**Mean**	**SD**	**Mean**	**SD**	**Mean**	**SD**	***p***	
Experimental group	91.43	1.21	75.34	1.34	89.36	1.46	0.042	C1 > C2^*^C3 > C2^*^
Control group	80.36	1.71	75.29	1.92	79.10	2.58	0.131	

* *p < 0.05*.

## Discussion

In this study, we apply educational data mining to explore the relationship between students' viewing behaviors and performance in accessing learning materials for flipped classrooms. The experiment was conducted to answer the research questions.

For question 1, the results of the experiment show significant differences in learning performance between the experimental group and the control group. In the control group, the students' SD was 10.243, which is a large gap. This result may show that most students are unable to complete flipped classroom tasks with the learning management system. When students preview learning materials that use the learning management system, they encounter difficulties in reading and are unable to use the learning management system to seek assistance from teachers or classmates; therefore, the students are unable to complete the flipped learning tasks. For the experimental group, this result may represent similar qualifications, and the gap in learning achievements may be closer after flipped classrooms. Therefore, the social media platform Facebook may be an effective way to help students complete the learning material preview tasks in the preclass asynchronous activity of flipped classrooms. This result is similar to previous studies; for example, Lai and Hwang (2016) found that students who watched learning materials through Facebook recalled a significantly greater amount of content from the learning materials than students who watched the learning materials through a learning management system.

For question 2, the results of the experiment show that there were no significant differences in the system user acceptance between the experimental group and the control group. These results indicate that the two groups did not have a significant difference on any item of system user acceptance. Next, we applied the paired sample *t*-test to the experimental and control groups to analyze the usefulness and ease of use. For usefulness, the *t*-test shows that there was no significant difference between the experimental group (*N* = 27, mean = 4.163, *SD* = 0.493) and the control group (*N* = 29, mean = 3.832, *SD* = 0.571), *t* = 0.532, *p* = 0.642, which indicates that there was no difference in the system usability of the two groups. Regarding ease of use, the results show that the two groups reached a significant difference (*t* = −2.136, *p* = 0.024 < 0.05) and that the average score was higher for the experimental group (*N* = 27, mean = 4.292, *SD* = 0.532) than for the control group (*N* = 29, mean = 3.623, *SD* = 0.482). This result may indicate that the students in the experimental group more easily used their system than the students in the control group. Therefore, using different systems with the experimental and control groups for flipped classrooms influenced the ease of use and affected the system user usability. Previous studies have found similar results. For example, Hou et al. (2013) found that students considered Facebook's user interface to be the most intuitive and easiest to use and more convenient than the learning management systems that were used by control group students. Lin et al. (2014) suggested that the use of Facebook during flipped classrooms could reduce the burden on students to operate the system. Students can thus quickly become proficient at lessons and read learning materials online by using educational systems with convenience anywhere, anytime. Therefore, the students' response to using Facebook in flipped classrooms was positive and generally acceptable.

For question 3, this study applies educational data mining analysis based on four indicators (viewing time, active viewing time, viewed amount, and actively viewed amount). The students in the experimental and control groups can be categorized into three different groups, namely, the actively engaged group (cluster 1, C1), the engaged viewing group (cluster 2, C2), and the long-term engagement group (cluster 3, C3). These results indicate that the students in the experimental and control groups exhibited distinct patterns while using different systems in flipped classrooms. The findings and discussions below are similar to those of several studies (Heffner and Cohen, 2005; Brooks et al., 2014; Lin et al., 2014; Park et al., 2017).

First, the students in the engaged viewing group spent a shorter viewing time than the students in the actively engaged group. Moreover, the students in the engaged viewing group spent significantly more time actively viewing and had a more actively viewed amount than the students in the actively engaged group. Although the differences were not significant, the results may indicate that the students in the engaged viewing group focused on the learning materials more than the students in the other groups. Therefore, the students in the engaged viewing group left the learning materials less frequently, and they rarely answered questions correctly in flipped classrooms. Park et al. (2017) found similar results: students rarely preread the learning materials before class. Lin et al. (2014) indicated that 1/3 of the students in their study were marked as minimal users of multimedia material viewing. This finding shows that students only want to inform their teachers that they have logged in and operated the system instead of engaging and completing all activities.

Second, the actively engaged group of students left the learning material frequently when the material was being viewed; thus, the students in the actively engaged group exhibited longer viewing times but shorter active viewing times and actively viewed lengths. The study is similar to Lin et al. (2014), whose students engaged actively with learning materials on Facebook with a medium frequency and used other learning tools more frequently; these behaviors are highly associated with flipped classrooms.

Third, the students in the long-term engagement group spent most of their time creating notes when viewing the learning materials, spent a large amount of time referring to the teacher's examples, and answered questions correctly while watching the learning materials repeatedly. Thus, the students in the long-term engagement group spent either a longer amount of time or watched more of the learning materials than the other groups. These observations may indicate that the students in the long-term engagement group regulated their behaviors to complete the flipped learning tasks by referring to other resources. In contrast, the other groups of students participated in this activity solely to complete the flipped learning tasks. This finding is similar to Lin et al. (2014). Their results may indicate that 1/2 of the students who were labeled long-term engagement users on Facebook viewed learning materials with a high frequency.

Finally, Kruskal–Wallis and Mann–Whitney U tests were used to measure the learning achievements among the actively engaged group, the engaged viewing group, and the long-term engagement group. The results show that the students' learning performance was significantly different in the experimental and control groups among the three clusters. These results may indicate that the behaviors of viewing the learning materials were associated with learning achievements in the experimental group. In particular, the students in the long-term engagement group and the actively engaged group gained significantly higher achievement than the students in the engaged viewing group. This indicates that the students who spent more time and effort on watching behaviors had better learning achievements. The results are similar to the findings of Heffner and Cohen (2005). Their results found that the amount of time that students spent viewing learning materials was positively correlated with their learning performance.

## CONCLUSION

In this paper, we apply educational data mining to explore the relationship between students' viewing behaviors in accessing learning materials and their performance in flipped classrooms. By conducting a two-group experimental design, we analyze the differences in the system user acceptance and learning performance between the two groups. Regarding viewing behaviors, our systems recorded students' operating processes, which helped us further explore the differences in the system user acceptance and learning performance between the two groups.

In the analysis of learning performance, the results indicate a significant difference between the experimental and control groups, with the mean of the experimental group being higher than the mean of the control group. The experimental group students used the social media platform Facebook in flipped learning, and most students' learning results improved significantly (Lai and Hwang, 2016; Su and Chen, 2020).

In the analysis of system user acceptance, the results show that there was no significant difference between the two groups. The mean of the experimental group was higher than 4.0, which indicates that the experimental group students highly accepted the use of the social media platform Facebook for flipped learning. However, the mean of the control group was 3.7, which indicates that the control group students reluctantly accepted the use of the learning management system for flipped learning (Hou et al., 2013; Lin et al., 2014).

Regarding the analysis of viewing behaviors and learning performance, the results show that the students in the experimental and control groups almost completed the flipped learning tasks; however, their viewing behaviors were totally different. To explore the processes of viewing the learning materials by the two groups, we analyzed the four indicators of the viewing time, active viewing time, viewed amount, and actively viewed amount. We applied the clustering method to analyze the viewing behavior patterns of the three categories of students: cluster 1 included the actively engaged group of students; cluster 2 included the engaged viewing group of students; and cluster 3 included the long-term engagement group of students. Next, we adopted the Kruskal–Wallis test to analyze the three categories of students. The results reveal significant differences among the three categories in the two groups. According to the results of the Mann–Whitney U test, there was a significant difference between clusters 1 and 2 and between clusters 2 and 3 in the experimental group. Additionally, in the experimental group, clusters 1 and 3 scored higher than cluster 2 in terms of learning performance. The findings indicate that the viewing behaviors and learning performance of the students in the experimental group differed significantly (Heffner and Cohen, 2005; Brooks et al., 2014; Lin et al., 2014; Park et al., 2017; Su et al., 2019, 2020).

According to the above findings and discussion, the learning achievements of students may be improved by using the social media platform Facebook for students to actively engage in flipped classrooms. Teachers and educators who are interested in applying appropriate educational tools can enhance student engagement by using Facebook with flipped classrooms. Therefore, the study provides insights that may be useful for understanding how students engage in flipped classrooms with the social media platform Facebook.

The limitation of this study is the small number of participants. As mentioned above, only 56 students participated in our course, which makes it difficult to generalize our study results. Therefore, in future work, we should increase the sample size to gain more generalizable conclusions.

## Data Availability Statement

The original contributions presented in the study are included in the article/supplementary material, further inquiries can be directed to the corresponding authors.

## Ethics Statement

Ethical review and approval was not required for the study on human participants in accordance with the local legislation and institutional requirements. Written informed consent for participation was not required for this study in accordance with the national legislation and the institutional requirements.

## Author Contributions

The authors contributed equally to the conception of the idea, implementing and analyzing the experimental results, and writing the manuscript. All authors read and approved the final manuscript.

## Conflict of Interest

The authors declare that the research was conducted in the absence of any commercial or financial relationships that could be construed as a potential conflict of interest.
